# Hypoxia-driven ncRNAs in breast cancer

**DOI:** 10.3389/fonc.2023.1207253

**Published:** 2023-07-31

**Authors:** Hashim H. Al-Zuaini, Kashif Rafiq Zahid, Xiangyan Xiao, Umar Raza, Qiyuan Huang, Tao Zeng

**Affiliations:** ^1^ Pharmacy Department, Al-kut University College, Kut, Iraq; ^2^ Department of Medical Laboratory, Affiliated Hospital of Guangdong Medical University, Zhanjiang, China; ^3^ Department of Radiation Oncology, Melvin and Bren Simon Comprehensive Cancer Center, Indiana University School of Medicine, Indianapolis, IN, United States; ^4^ Department of Medical Laboratory, School of Laboratory Medicine and Biotechnology, Southern Medical University, Guangzhou, China; ^5^ Department of Biological Sciences, National University of Medical Sciences (NUMS), Rawalpindi, Pakistan; ^6^ Department of Clinical Biobank Center, Zhujiang Hospital, Southern Medical University, Guangzhou, China

**Keywords:** hypoxia, HIFs, breast cancer, ncRNA, miRNAs, lncRNAs, circRNAs

## Abstract

Low oxygen tension, or hypoxia is the driving force behind tumor aggressiveness, leading to therapy resistance, metastasis, and stemness in solid cancers including breast cancer, which now stands as the leading cause of cancer-related mortality in women. With the great advancements in exploring the regulatory roles of the non-coding genome in recent years, the wide spectrum of hypoxia-responsive genome is not limited to just protein-coding genes but also includes multiple types of non-coding RNAs, such as micro RNAs, long non-coding RNAs, and circular RNAs. Over the years, these hypoxia-responsive non-coding molecules have been greatly implicated in breast cancer. Hypoxia drives the expression of these non-coding RNAs as upstream modulators and downstream effectors of hypoxia inducible factor signaling in the favor of breast cancer through a myriad of molecular mechanisms. These non-coding RNAs then contribute in orchestrating aggressive hypoxic tumor environment and regulate cancer associated cellular processes such as proliferation, evasion of apoptotic death, extracellular matrix remodeling, angiogenesis, migration, invasion, epithelial-to-mesenchymal transition, metastasis, therapy resistance, stemness, and evasion of the immune system in breast cancer. In addition, the interplay between hypoxia-driven non-coding RNAs as well as feedback and feedforward loops between these ncRNAs and HIFs further contribute to breast cancer progression. Although the current clinical implications of hypoxia-driven non-coding RNAs are limited to prognostics and diagnostics in breast cancer, extensive explorations have established some of these hypoxia-driven non-coding RNAs as promising targets to treat aggressive breast cancers, and future scientific endeavors hold great promise in targeting hypoxia-driven ncRNAs at clinics to treat breast cancer and limit global cancer burden.

## 1 Introduction

### 1.1 Breast cancer

Despite the substantial improvements in cancer prevention, diagnosis, and treatment over the last few decades, 19.3 million new cases and almost 10 million cancer associated deaths were recorded globally in 2020 (1). Breast cancer is now the most prevalent malignancy worldwide and is the leading cause of cancer related mortality among women (2). The heterogeneous nature of breast cancer has led to its classification into different subtypes based on both clinical and molecular characteristics, with each subtype exhibiting varying biological and pathological traits as well as distinct clinical outcomes (3). In clinical practice, immunohistochemical quantification of Estrogen Receptor (ER), Progesterone Receptor (PR) and Human Epidermal Growth Factor Receptor 2 (HER2) is the gold standard to classify breast cancer into hormone receptor positive (HR+), HER2 positive (HER2+) and triple negative breast cancer (TNBC), whereas transcriptional profiling stratifies breast cancer into five major subtypes, i.e. luminal A, luminal B, HER2 over-expression, basal, and normal-like tumors primarily overlapping clinical subtypes (4). For instance, luminal tumors overlap with the HR+ clinical subtype. These tumors grow slowly but often relapse, and are treated with a combination of endocrine- and chemo-therapy (5). HER2+ breast cancer is relatively aggressive, with a poor prognosis and treatment options revolving around targeting HER2 along with chemotherapy (6). Basal or TNBC represent the most aggressive subtype of breast cancer, with chemotherapy as sole treatment option due to lack of the expression of targetable receptors (7). Unfortunately, therapy resistance is inevitable due to cellular heterogeneity of breast cancer, and conventional treatment options often fall short of tackling almost all aggressive breast cancer cases (8). On top of that, distant metastatic spread further complicates the scenario and is considered incurable, as almost 90% of breast cancer associated deaths are due to metastatic disease (9). Therefore, discovering new drugable targets, developing potent therapies, and identifying innovative therapeutic strategies are indispensable for breast cancer.

### 1.2 Hypoxia and cancer

**Figure 1:**
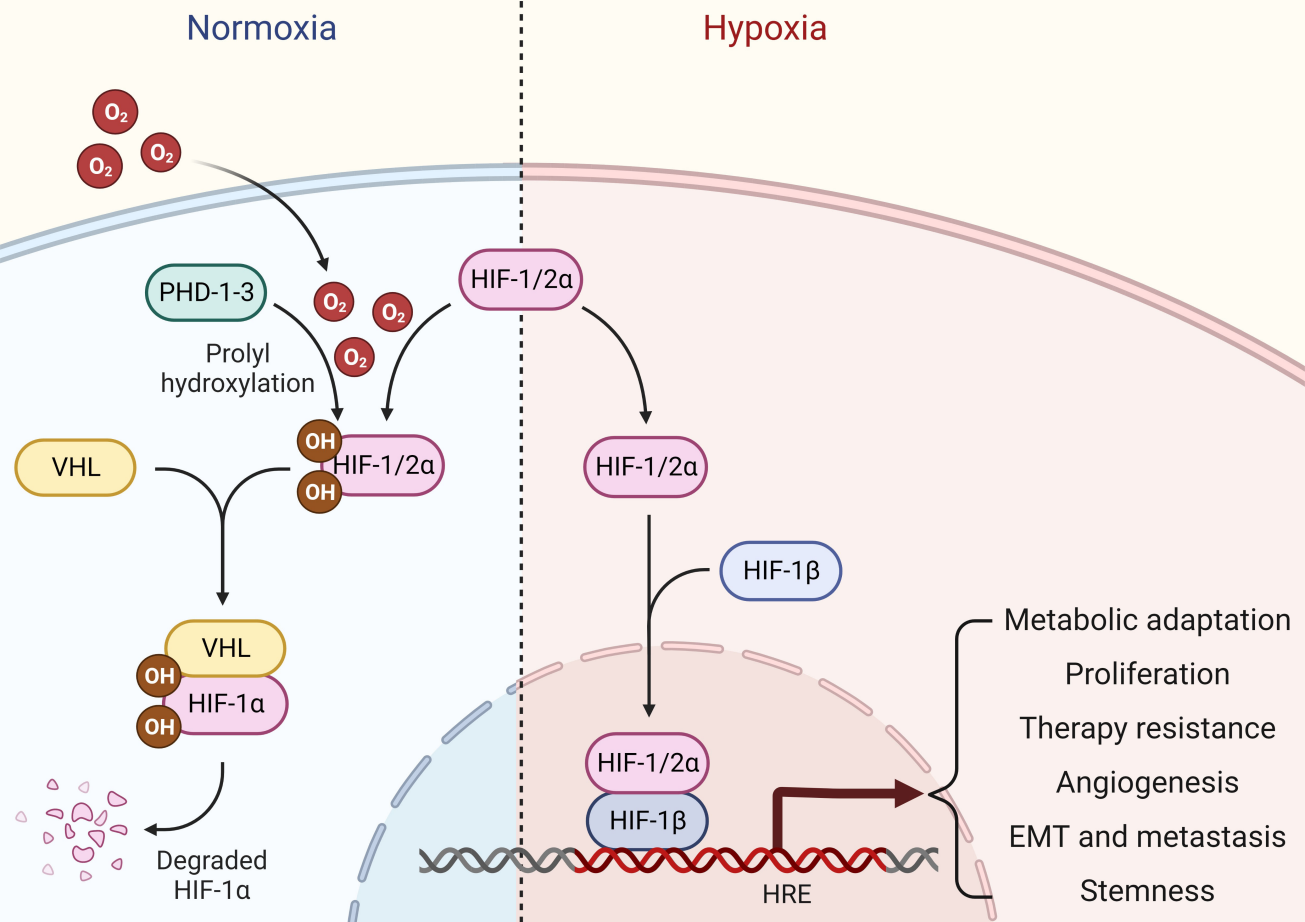
Hypoxia signaling. Under normal oxygen availability, HIF1/2α are hydroxylated by PHDs, which are then ubiquitinated and undergo proteasomal degradation in an E3 ubiquitin ligase VHL-dependent manner. Upon oxygen deprivation, HIF1/2α escapes degradation and binds to form heterodimeric HIF transcription factors with constitutively expressed and oxygen insensitive HIF1β subunit. This complex is then translocated into the nucleus, where it binds to HREs in the promoter regions and stimulates gene transcription that aids cancer cells for metabolic adaptations, proliferation, therapy resistance, angiogenesis, EMT, metastasis and stemness.

Oxygen is the driving force behind cellular metabolism, homeostasis, and survival. During the initial stages of oncogenic transformation, oxygen and nutrients are still readily available to transformed cells because they are in close proximity to normal tissue vasculature in their surroundings (10). As these cells progress to form tumors, low oxygen tension, or hypoxia, starts to prevail in almost all solid tumors, especially in the central core of the mass (11). As a result of uncontrolled proliferation outgrowing surrounding vasculature, oxygen levels markedly plunge in these hypoxic regions, leading to changes in the pH gradient towards an acidic environment (12). Although transformed cells tend to die in the beginning within the hypoxic core as oxygen and glucose deprivation trigger necrosis due to a drop in cellular adenosine triphosphate (ATP) levels (13), these cancer cells adapt quickly to changing microenvironment and orchestrate a hypoxic signaling response to favor cancer cell survival, tumor progression, therapy resistance, metastasis, and cancer stemness overtime (14, 15). The classical hypoxic response involves the stabilization of oxygen-sensitive molecular mediators called hypoxia-inducible factors (HIFs). Under normoxia, HIFs (HIF1α, HIF2α, HIF3α) are continuously hydroxylated by prolyl-4-hydroxylases (PHDs), and these hydoxylated HIFs are under selective pressure to be degraded by an E3 ubiquitin ligase called Von Hippel-Lindau (VHL). Upon oxygen deprivation, HIF1α escapes degradation and binds to form heterodimeric HIF1 transcription factor with constitutively expressed and oxygen insensitive HIF1β subunit. This complex is then translocated into the nucleus where it binds to hypoxia-responsive elements (HREs) in the promoter regions and transcribes hundreds of genes involved in multiple signaling pathways controlling cancer promoting cellular mechanisms such as proliferation, apoptosis, extracellular matrix remodeling, epithelial-to-mesenchymal transition (EMT), and angiogenesis (16). Alternatively, HIF2α can partner with HIF1β upon oxygen deprivation and form the HIF2 transcription factor. Notably, HIF1 and HIF2 transcription factors share multiple common targets alongside unique sets of genes that they individually transcribe (17) (**Figure 1**). Alternatively, HIF3α may compete with HIF1α and HIF2α to bind with HIF1β, thereby inhibiting HIF-dependent gene regulation (18). In addition, hypoxia may drive a non-classical response as well by regulating gene expression through HIF-independent mechanisms (19, 20). Given its central role in cancer progression, targeting hypoxia and HIF-mediated signaling pathways provides excellent therapeutic option for treating solid tumors such as breast cancer in clinical settings.

### 1.3 ncRNAs and cancer

**Figure 2:**
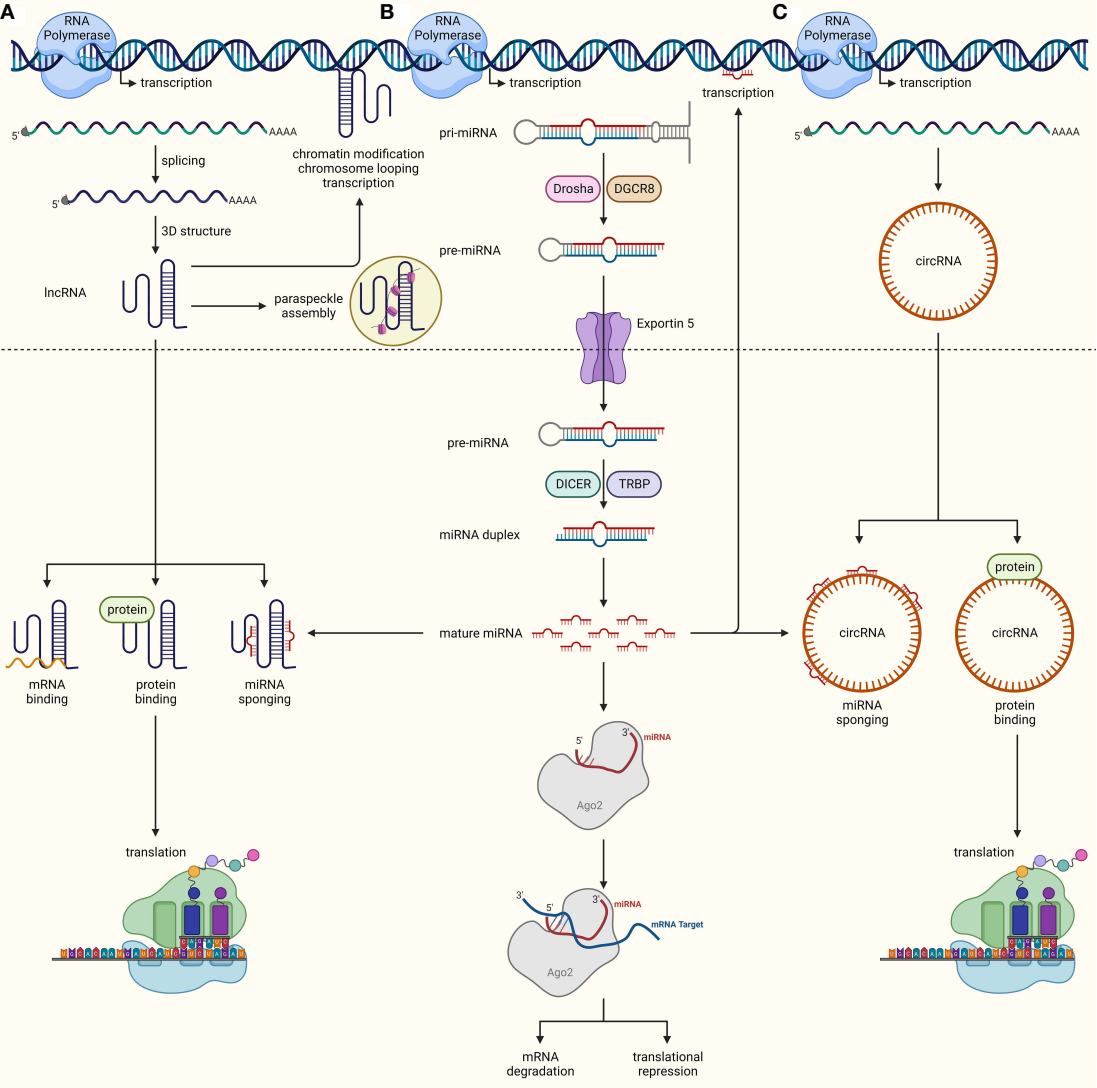
Biogenesis and functions of ncRNAs. **(A)** lncRNAs are transcribed from the genome similarly to mRNAs, undergo splicing, and adopt complex 3D structures upon maturation inside the nucleus. They can either work inside the nucleus to regulate chromatin modifications, chromosomal looping, DNA transcription, and paraspeckle formation, or they can be exported out to the cytoplasm, where they interact with mRNAs, proteins, and miRNAs, and regulate protein translation and modifications, as well as compete with miRNAs by sponging them off their targets. **(B)** miRNAs are transcribed as pri-miRNAs from the genome, which are processed into pre-miRNA by DROSHA and DGCR8. These pre-miRNA are then exported out into the cytoplasm through XPO5, where they are transformed into miRNA-duplexes through cleavage by DICER. One strand of duplex is then loaded into RISC, where its seed region binds complementarily to target mRNA, leading to mRNA degradation or translational repression. miRNAs may also aid in transcription or interact with other ncRNAs. **(C)** CircRNAs mature through diversified biogenesis mechanisms such as exon-skipping, intron debranching, intron pairing, and dimerization of RNA binding proteins. Similar to lncRNAs, circRNAs can also modulate protein translation and function through direct interactions and miRNA inhibition by sponging them off their target.

Over the last two decades, scientists have discovered the great virtues of the non-coding genome in regulating gene expression. It all started with the identification of small (19-24 nucleotides in length) ncRNAs called microRNAs (miRNAs). These tiny structures are transcribed from genome as primary miRNA (pri-miRNA) which are then processed inside the nucleus by DROSHA and DGCR8 into precursor miRNA (pre-miRNA). Once exported to the cytoplasm by exportin 5 (XPO5), pre-miRNAs are further cleaved by DICER to become mature miRNAs which can preferentially bind to the 3’UTR of their target mRNAs in the miRNA-induced silencing complex (RISC) leading to translational repression or mRNA degradation depending upon miRNA-mRNA binding synergy (21, 22). miRNAs are involved in every cellular and biological process, as they are estimated to regulate almost 60% of the mammalian genome (23). In addition to their classical mRNA targeting function, miRNAs may maintain cellular homeostasis in other ways, such as by inhibiting nuclear ncRNAs, binding and inhibiting proteins, inhibiting mitochondrial transcripts, coding for peptides, triggering transcription, activating the translation of mRNAs, and activating Toll-like receptors (24). Long ncRNAs (lncRNAs) denote another class of regulatory ncRNAs comprising RNA molecules of around 200 nucleotides in length that are not translated into proteins. LncRNAs are distributed throughout the genome and are transcribed and processed in similar fashion to mRNAs, though they tend to form multifaceted 3D structures with the flexibility to rapidly change and perform multiple functions (25). LncRNAs may work in a *cis* (in proximity to their transcription site) or *trans* (at distant sites) fashion. Their known functions include, but are not limited to, chromatin modification, chromosomal looping, gene transcription, binding to mRNAs and proteins, interacting with other ncRNAs, protein translation, and paraspeckle formation (26). The third major class of regulatory ncRNAs is circular RNAs (circRNAs) which are joined end-to-end to form loops. They are generated through diversified biogenesis mechanisms such as exon-skipping, intron debranching, intron pairing, and dimerization of RNA binding proteins. CircRNAs play a role in cellular homeostasis by sponging miRNAs, controlling protein translation and binding proteins to regulate their functions (27) (**Figure 2**). Deregulated expression of miRNAs, lncRNAs, and circRNAs in cancer cells links these ncRNAs to different hallmarks of cancer, such as uncontrolled proliferation, resistance to cell death, invasion and metastasis, angiogenesis, immune evasion, and cancer stemness (28). Notably, unlike most protein-coding genes, a ncRNA may exhibit cancer promoting or inhibiting functions in tissue- and context-dependent manners, thereby adding another level of complexity to our understanding of the true nature of ncRNAs in cancer (29). Recently, multiple other types of ncRNAs such as piwi-interacting RNAs (piRNAs), small nuclear RNAs (snRNAs), and small nucleolar RNAs (snoRNAs), have also been explored to perform key regulatory mechanisms (30), though understanding of their role in cancer biology is still limited.

Here, we aimed to comprehensively review the role of hypoxia-driven ncRNAs in breast cancer and their therapeutic potential in clinics. In this context, we have first discussed the molecular mechanisms through which hypoxia drives the expression of different ncRNAs as upstream modulators and downstream effectors of hypoxia inducible factor (HIF) signaling in favor of breast cancer. In addition, we have also summarized the contribution of these ncRNAs in orchestrating aggressive hypoxic tumor environment by regulating cancer associated cellular processes such as proliferation, evasion of apoptotic death, extracellular matrix remodeling, angiogenesis, migration, invasion, EMT, metastasis, therapy resistance, stemness, and evasion of the immune system in breast cancer. We have reviewed the interplay between hypoxia-driven ncRNAs as well as feedback and feedforward loops between these ncRNAs and HIFs in breast cancer. Later, we have presented the current clinical implications of hypoxia-driven ncRNAs as prognostic and diagnostic markers in breast cancer. Lastly, we have discussed the current developments and challenges in therapeutic strategies as well as future prospects for targeting hypoxia-driven ncRNAs in breast cancer.

## 2 Hypoxia-driven ncRNAs in breast cancer

In addition to regulating a plethora of protein-coding genes, hypoxia may regulate the expression of different ncRNAs both in HIF-signaling-dependent and independent manners. The underlying molecular mechanisms include transcriptional regulation as well as post-transcriptional regulation such as editing, modification, maturation, stability, localization, and transport of these ncRNAs (31–34). For instance, changes in miRNA editing patterns, particularly if miRNAs are edited in their seed regions, may alter the whole transcriptome, as a single miRNA can target multiple mRNAs at the same time (35). Recently, hypoxia has been suggested as a key external stimulus that can alter the whole transcriptomic profile of cells by inducing miRNA editing in seed regions (36). Hypoxia may affect the processing of miRNA transcripts into mature miRNAs in breast cancer cells by downregulating DICER, the key enzyme involved in miRNA biogenesis (37, 38). Alternatively, epidermal growth factor receptor (EGFR) binds to argonaute 2 (AGO2) and phosphorylates it at Tyr 393 under hypoxia, thereby disrupting the interaction between AGO2 and DICER and resulting in impaired miRNA maturation. This leads to the evasion of multiple oncogenes from miRNA inhibition, thereby contributing to invasion and metastasis in breast cancer (39). In addition, the expression of multiple classes of ncRNAs, including miRNAs, lncRNAs, circRNAs, and snRNAs, is fine-tuned by hypoxia to facilitate cancer progression (34). This section summarizes the contributory roles of ncRNA as upstream modulators and as downstream effectors of hypoxia in orchestrating an aggressive hypoxic tumor environment in breast cancer.

### 2.1 ncRNAs as upstream modulators of HIF-signaling in breast cancer

**Figure 3:**
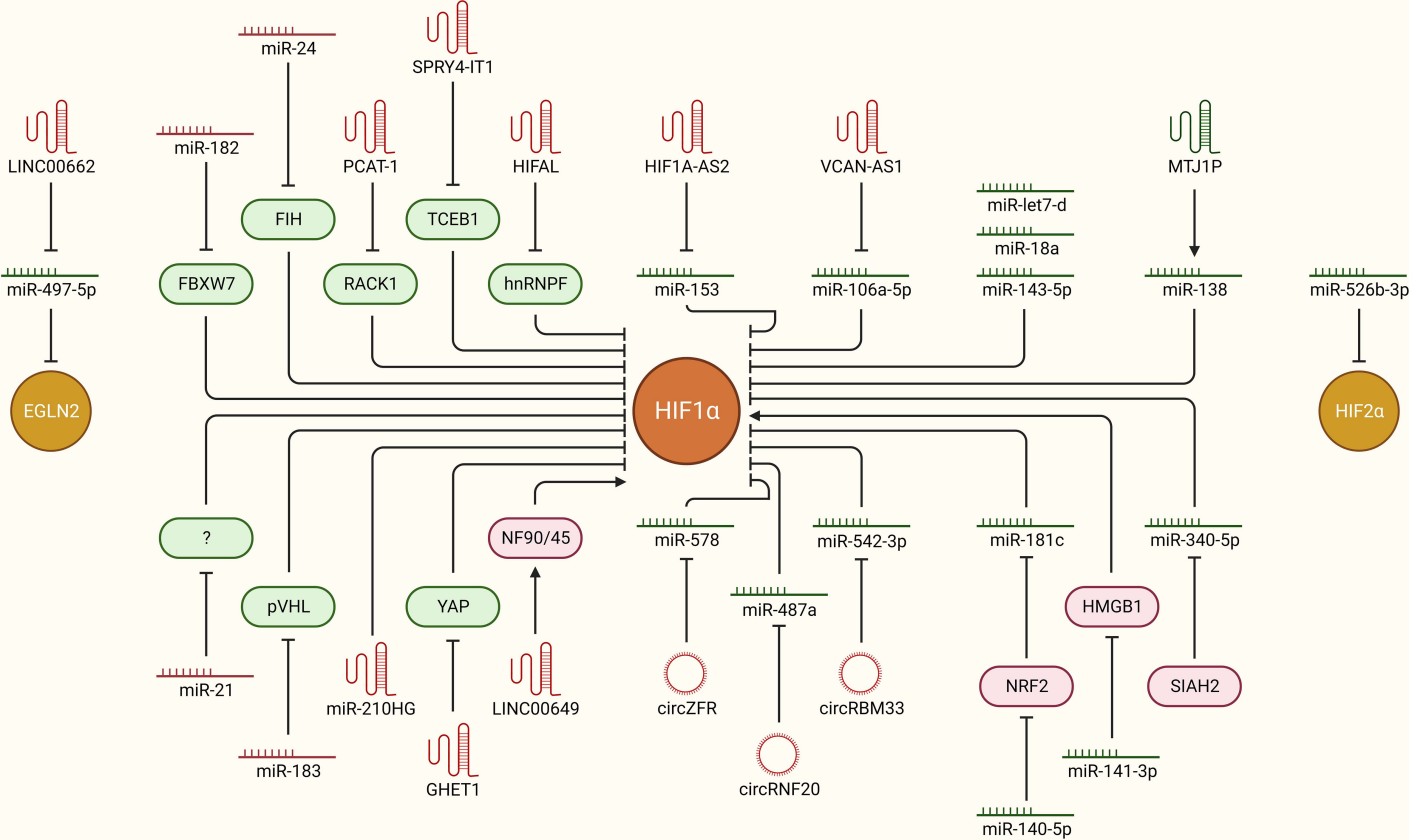
ncRNAs as upstream modulators of HIF-signaling in breast cancer. Hypoxia**-**driven ncRNAs are implicated in regulating the expression of HIFs to modulate downstream oncogenic signaling pathways in breast cancer. The mechanistic regulation of HIFs downstream of different ncRNAs (miRNAs, lncRNAs, and circRNAs) in breast cancer is summarized. Green: anti-tumor, Red: pro-tumor, arrowhead: activation, blockhead: inhibition.

The overall hypoxic phenotype is mainly coordinated through oxygen deprivation-driven HIF-dependent signaling. How HIFs are stabilized and activated in the first place to orchestrate the hypoxic tumor microenvironment is of key importance. Multiple ncRNAs, including miRNAs, lncRNAs and circRNAs, are now implicated in the regulation of HIFs upon oxygen deprivation *via* direct or indirect interactions in breast cancer (**Figure 3**). For instance, miR-182 aids in hypoxia-driven cancer progression by directly targeting the E3 ubiquitin-protein ligase FBXW7, which preferentially tags HIF1 for degradation. Furthermore, upregulated miR-182 under hypoxia induces vascular endothelial growth factor A (VEGFA) expression downstream of HIF1-signaling to promote angiogenesis in breast tumors (40). miR-24 regulates HIF1α expression specifically in breast cancer stem cells (CSCs) and promotes cancer stemness. Mechanistically, it targets factor inhibiting HIF1α (FIH1), which is a negative regulator of HIF1α, leading to increased expression of HIF1α (41). A well-established oncogenic miRNA, miR-21, may also upregulate HIF1α expression and promote EMT-like features and stemness in breast cancer cells, though the molecular link between miR-21 and elevated HIF1α expression is not established yet (25). Oncoprotein hepatitis B X-interacting protein (HBXIP) escalates tumorigenesis by enhancing HIF1α expression in multiple ways. It targets VHL and disassociates it from HIF1α to stabilize HIF1α expression. As a result, HIF1α may promote miR-183/96/182 cluster expression, out of which miR-183 can also target VHL mRNAs to further inhibit its expression and constitutively promote HIF1α expression and associated cancer progression in a feedback loop mechanism. In addition, these miRNAs may also target key regulators of glucose metabolism, such as synthesis of cytochrome C oxidase 2 (SCO2) and pyruvate dehydrogenase alpha 1 (PDHA1), in favor of cancer progression (42). These findings suggest that multiple oncogenic miRNAs indirectly regulate HIF signaling, and targeting these molecules holds promise to limit hypoxic TME and cancer progression.

LncRNA prostate cancer-associated transcript-1 (PCAT-1) is induced upon hypoxia in breast cancer cells and stabilizes HIF1α by directly interacting with the receptor of activated protein C kinase-1 (RACK1) protein and preventing RACK1-mediated degradation of HIF1α. Unsurprisingly, PCAT-1 is upregulated in breast cancer patients and is also associated with clinical parameters such as grade, tumor size, and poor clinical outcome (43). Similarly, high expression of the lncRNA SPRY4 intronic transcript 1 (SPRY4-IT1) is associated with cancer aggressiveness and poor outcome in breast cancer. It promotes cancer cell metastasis by upregulating HIF1α expression. Mechanistically, NF-kB/p65 transactivates SPRY-IT1, which inhibits transcription elongation factor B-polypeptide 1 (TCEB1) expression by promoting Staufen 1 (STAU1) mediated decay of TCEB1 mRNA transcripts. In turn, the activity of E3 ubiquitin ligases decreases, which results in elevated HIF1α expression, leading to increased cancer progression and metastasis (44). HIF-1α anti-sense lncRNA, HIFAL, is also critical in maintaining a hypoxic tumor environment by promoting HIF1α expression in a feedback loop. It first induces prolyl hydroxylation of pyruvate kinase 2 (PKM2) by recruiting PHD3 and later aids in translocating the PKM2/PHD3 complex into the nucleus *via* binding heterogeneous nuclear ribonucleoprotein F (hnRNPF). Once inside the nucleus, the PKM2/PHD3 complex enhances HIF-1α transactivation, which in turn induces HIFAL transcription to maintain the feed-forward loop. Notably, HIFAL expression is also validated to be associated with an aggressive phenotype and poor clinical outcome in breast cancer patients (45). Higher expression of MIR210HG lncRNA is also associated with poor clinical outcome in breast cancer patients. At the molecular level, hypoxia induced MIR210HG stabilizes HIF1α expression by directly binding its 5’UTR. As a result, increased HIF1α expression promotes glycolytic metabolism and tumorigenic potential in TNBC cells (46). Hypoxia induced lncRNA gastric carcinoma proliferation enhancing transcript 1 (GHET1) is highly expressed in TNBC tissues and is associated with extensive cell proliferation, invasion, and glycolysis. The downstream effects of GHET1 are attributed to the induction of HIF1α expression and activation of Hippo/Yap signaling pathway through the promotion of nuclear translocation of Yes1 associated transcriptional regulator (YAP) (47). LINC00649 stabilizes HIF1α expression and promotes tumor growth and metastasis in breast cancer. Mechanistically, it stabilizes HIF1α mRNA by interacting with the nuclear factor 90 (NF90)-NF45 complex and upregulates HIF1α expression (48). These findings highlight that multiple lncRNAs boost HIF-signaling directly or indirectly upon hypoxia, and targeting these lncRNA can aid to limit cancer progression.

Certain lncRNAs and circRNAs can alter cellular transcriptomic profiles and associated phenotype through sponging off miRNAs from their mRNA targets, leading to enhanced expression of a set of genes. So far, a few lncRNAs and circRNAs have been reported to regulate hypoxia through such mechanism in breast cancer. For instance, miR-153 inhibits angiogenesis in breast cancer tissues by directly targeting HIF1α and angiopoietin (ANG1), thereby suppressing the migration and invasion of cancer cells (49). Interestingly, in order to fine-tune the angiogenesis in TNBC, hypoxia-driven ER stress also induces miR-153 expression in an endoribonuclease IRE1-like protein (IRE1A)-X-box binding protein 1 (XBP1) complex-dependent manner, where XBP1 directly binds to miR-153 host gene protein tyrosine phosphatase receptor type N (PTPRN) and promotes its transcription. In return, miR-153 directly targets the HIF1α 3’ UTR and inhibits downstream VEGFA mediated angiogenesis (50). Unfortunately, this whole axis is balanced in favor of increased angiogenesis preferentially through hypoxia mediated induction of the lncRNA HIF1α-anti sense 2 (AS2), which sponges off miR-153 from HIF1α mRNA transcripts (51). LncRNA versican (VCAN)-AS1 promotes breast cancer cell migration, invasion, and EMT by working as a competing endogenous RNA to inhibit miR-106a-5p, which cancer cell progression *via* targeting signal transducer and activator of transcription 3 (STAT3) and inhibiting the STAT3/HIF1α axis (52). LINC00662 sponges off miR-497-5p from the egl-9 family hypoxia inducible factor 2 (EglN2) and promotes breast cancer cell proliferation, migration, and invasion (53). CircZFR promotes breast cancer progression and metastasis by relieving HIF1α from being targeted by miR-578. It sponges off miR-578 from HIF1α. On the other hand, miR-578 promotes apoptosis by targeting HIF1α (54). CircRNF20 also plays a role in cancer progression through stabilizing HIF1α and downstream signaling in breast cancer cells. Mechanistically, it sponges off miR-487a from mRNAs of HIF1α, which results in HIF1α mediated transcription of hexokinase II (HK2) and promotes glycolysis and proliferation in cancer cells (55). Similarly, miR-542-3p works as a negative regulator of HIF1α, but unfortunately it is downregulated in breast cancer cells through a HIF1α-driven feed-back loop. Mechanistically, increased hypoxia in cancer tissue masks the miR-542-3p mediated inhibition of HIF1α by upregulating circRBM33 *via* transcriptional activation by HIF1α itself. Resultantly, increased HIF1α expression induces proliferation and promotes glycolysis in cancer cells (56). Overall, multiple lncRNAs and circRNAs work as positive regulators of HIF-signaling by sponging of miRNAs, and targeting these ncRNAs in clinics provide a great avenue to limit hypoxic TME and associated tumor progression in breast cancer patients.

miRNA-mediated tightly controlled regulation of HIFs and other associated signaling factors is key determinant in bringing about the molecular changes behind the normoxia-hypoxia shift upon oxygen deprivation. For instance, HIF1α is a direct target of miR-143-5p, which inhibits tumorigenesis, cancer proliferation, and progression by suppressing HIF1α expression at the post-transcriptional level and solute carrier family 2 member 1 (GLUT1) pathway downstream of HIF1α signaling. Unfortunately, miR-143-5p expression is decreased in breast cancer clinical samples compared to adjacent healthy tissues, suggesting that hypoxia may overcome miR-143-5p induced HIF1α downregulation, though the underlying mechanism is still unexplored (57). Another miRNA, let-7d, also regulates HIF1α expression in breast cancer cells. Loss of let-7d and gain of HIF1α activity are in concordance in patient datasets and clinical breast cancer samples. Increased HIF1α expression and activity due to loss of let-7d leads to elevated expression of platelet-derived growth factors (PDGFA/B), resulting in increased brain metastasis of breast cancer cells (58). Expression of miR-18a from the miR17HG family is downregulated in a cell line-based experimental TNBC model *in vitro* and *in vivo*. Upregulation of this miRNA inhibits HIF1α expression through direct targeting and suppresses tumor growth at primary sites as well as metastasis to the lungs (59). Under hypoxic conditions, nuclear factor erythroid 2-like-2 (NRF2) aids in stabilizing HIF1α expression and subsequent downstream signaling by inhibiting miR-181c, which directly targets HIF1α. This supports cancer cells’ metabolic adaptation *via* heightened glycolysis, aiding in proliferation and survival (60). Alternatively, miR-140-5p may suppress hypoxia-driven tumor growth, angiogenesis, invasion, and metastasis *via* targeting NRF2, but its expression is downregulated upon hypoxia in breast cancer cells (61). High‐mobility group box protein 1 (HMGB1) works as a positive regulator of hypoxia by directly regulating HIF1α expression. This axis is fine-tuned by miR-141-3p, which post-transcriptionally inhibits HMGB1 expression upstream. Notably, miR-141-3p expression is low in breast cancer tissues and is associated with poor prognosis, but how hypoxia downregulates its expression in the first place is not yet established (62). LncRNA, Metallothionein 1 J, pseudogene (MT1JP), may suppress cancer cell proliferation and migration by transcriptionally activating miR-138, which directly targets HIF1α mRNA transcripts. Unfortunately, expression of both MTJ1P and miR-138 progressively downregulates as TNBC tumors advance towards an aggressive and hypoxic phenotype (63). Hypoxia-induced downregulation of miR-340-5p promotes EMT and metastasis in breast cancer cells by upregulating SIAH2 expression, which directly stabilizes HIF1α expression (64). In addition, miR-526b-3p may suppress hypoxia induced notch signaling and inhibit cancer cell stemness and chemoresistance by directly targeting HIF2α. This sensitizes cancer cells to paclitaxel treatment, as well as attenuates stem cell-like features (65). Although certain ncRNAs have the potential to counteract hypoxia by directly or indirectly regulating HIFs, these molecules are often downregulated in hypoxic, advanced stage breast cancers. Clinical activation of such axes may be fruitful in limiting hypoxia-driven tumor growth and progression in breast cancer.

### 2.2 ncRNAs as downstream effectors of hypoxia/HIF-signaling in breast cancer

**Figure 4:**
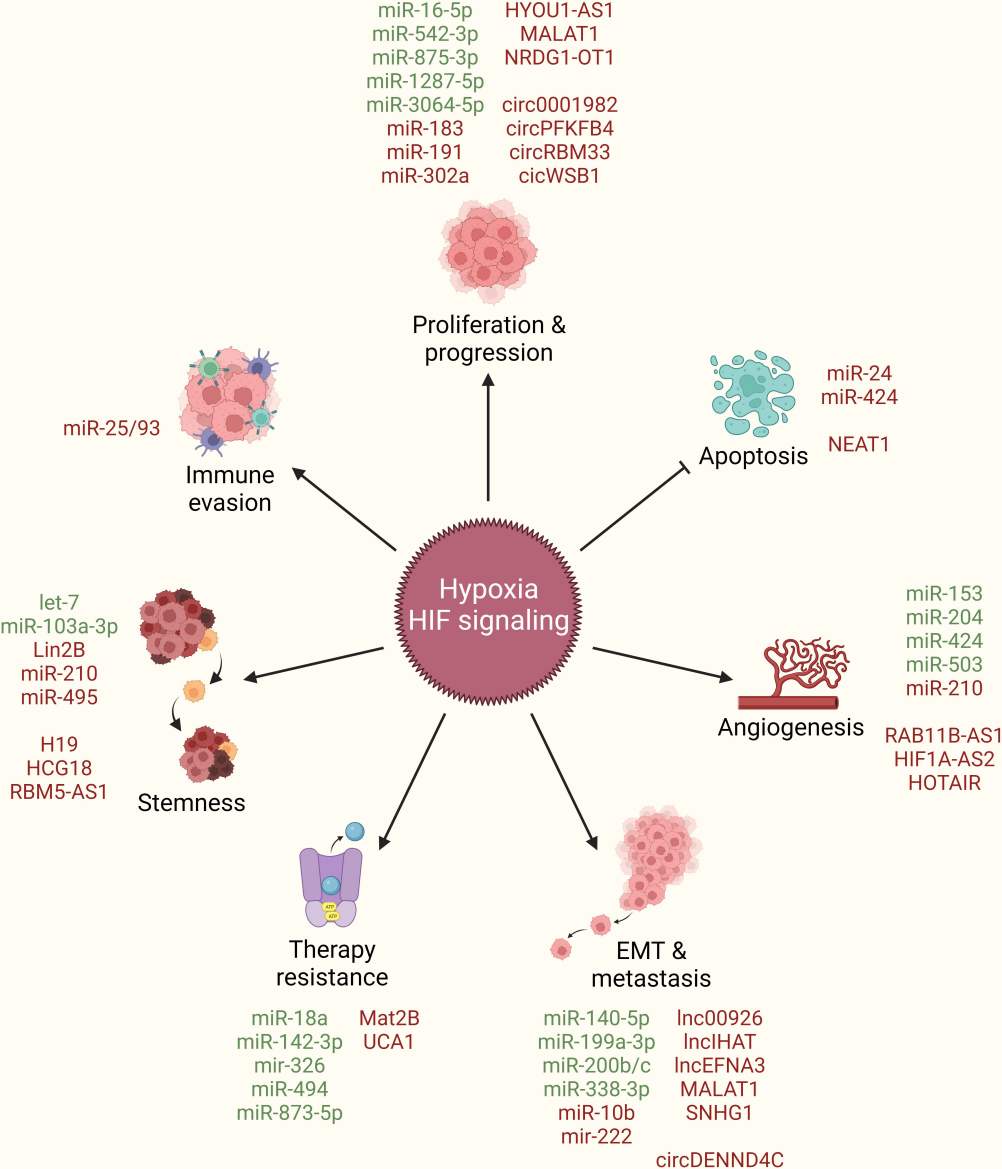
ncRNAs as downstream effectors of hypoxia/HIF-signaling in breast cancer. Hypoxia/HIF-signaling regulate the expression of different ncRNAs (miRNAs, lncRNAs, circRNAs) to promote cancer cell proliferation and progression, limit apoptotic cell death, induce angiogenesis, stimulate EMT and metastasis, resist therapeutic interventions, acquire stem cell-like features, and evade immune system. Green: Inhibited by hypoxia/HIF-signaling, Red: Activated by hypoxia/HIF-signaling, arrowhead: activation, blockhead: inhibition.

In addition to their above discussed role in boosting HIF-signaling and orchestrating hypoxic TME, multiple ncRNAs are also regulated as the downstream effectors of HIF-signaling to directly endorse breast cancer by taking part in promoting cancer cell proliferation and progression, limiting apoptotic cell death, inducing angiogenesis, stimulating EMT and metastasis, resisting therapeutic interventions, acquiring stem cell-like features, and evading immune system (**Figure 4**).

#### 2.2.1 Proliferation and progression

Hypoxia regulates the expression of multiple ncRNAs, including miRNAs, lncRNAs, circRNAs, through varying mechanisms to promote breast cancer proliferation and progression. For instance, hypoxia induces the expression of the pro-tumorigenic driver YTH N6-methyladenosine RNA binding protein F1 (YTHDF1), and promotes breast cancer cell proliferation through dual mechanisms; first, by promoting its transcriptional activation in HIF1α-dependent manner, and second, by inhibiting the miR-16-5p-mediated post-transcriptional repression of YTHDF1. In return, YTHDF1 promotes glycolysis by activating PKM2 expression and promotes cancer cell proliferation and migration (66). As mentioned earlier, HIF1α may promote miR-183/96/182 cluster expression in an HBXIP-dependent manner, out of which miR-183 can target VHL mRNAs and inhibit its expression, thereby constitutively promoting HIF1α-driven tumorigenesis and cancer progression in a feedback loop mechanism. In addition, these miRNAs may also target SCO2 and PDHA1 and promote glucose metabolism in favor of cancer progression (42). Hypoxia induces miR-191 expression in HIFs-dependent manner and promotes breast tumor aggressiveness. In turn, miR-191 targets the RNA binding protein HUR and indirectly upregulates transforming growth factor beta 2 (TGFβ2) expression, which leads to upbeat TGFβ signaling and increases the migration of cancer cells (67). Cancer cells tend to decrease proliferation and migrate towards excessive nutrient availability during the early stages of hypoxia. In this line, miR-491 tend to inhibit proliferation by targeting B-cell lymphoma-extra large (Bcl-xl), whereas miR-302a tends to promote cell migration by targeting C-X-C chemokine receptor 4 (CXCR4) expression in cancer cells upon hypoxia (68) though molecular link between HIF-signaling and induction of these miRNAs is not established so far.

The Hpoxia up-regulated 1 (HYOU1) gene is associated with poor clinical outcomes in breast cancer patients. Interestingly, the same gene locus expresses a lncRNA from the opposite strand named HYOU1-AS1, which aids in HYOU1 mediated TNBC cell proliferation and progression through competitively binding to heterogeneous nuclear ribonucleoprotein A1 (hnRNPA1), an RNA binding protein that may post-transcriptionally inhibit HYOU1 mRNA (69). HIF1α transcriptionally activates the lncRNA N-Myc downstream regulated 1-overlapping 1 (NDRG1-OT1) in breast cancer cells, which aids in cancer progression by destabilizing its parent gene NDRG1 both at transcriptional and post-translational levels. At the transcriptional level, it impairs promoter activity by increasing the binding of hnRNPA1 and limiting KH-Type Splicing Regulatory Protein (KHSPR) accumulation at promoter site. At post-translational level, it promotes degradation of NDRG1 *via* the ubiquitin proteasome pathway (70, 71). lncRNA NDRG1-OT1 may work as a miRNA sponge to particularly inhibit a tumor suppressor miRNA, miR-875-3p, and promote cancer cell proliferation, though functional targets at the downstream of the HIF1α/NDRG1-OT1/miR-875-3p axis have not been established so far (72). Metastasis associated lung adenocarcinoma transcript 1 (MALAT1) is a hypoxia-responsive lncRNA that promotes proliferation and migration in breast cancer cells. Interestingly, MALAT1 can be transcribed in both HIF1α- and HIF2α-dependent manners and stimulate tumor growth and migration by sponging off miR-3064-5p in breast cancer cells, though downstream functional targets of miR-3064-5p have not been identified (73).

Hypoxia promotes breast cancer proliferation and progression by inducing certain circRNAs as well. For instance, upon transcriptional activation through HIF1α, circPFKFB4 binds to damage specific DNA binding protein 1 (DDB1)/DDB2 and aids in cullin-ring ligase-4 (CRL4)-DDB2 ubiquitin ligase assembly, which in turn promotes cancer cell proliferation by specifically increasing the degradation of tumor suppressor p27 (74). circWSB1 expression correlates with poor prognosis and clinical outcome in breast cancer patients. It is also induced in a HIF1α-dependent manner and promotes cancer proliferation by destabilizing p53. It primarily targets ubiquitin specific peptidase 10 (USP10), which no longer deubiquitinates its targets, leading to p53 degradation (75). Hypoxia-driven HIF1α expression transcriptionally activates circRBM33, which aids in further enhancing HIF1a expression by masking miR-542-3p mediated inhibition of HIF1α. Resultantly, increased HIF1α expression induces proliferation through elevated glycolysis in cancer cells (56). Hypoxia upregulates circ0001982, which sponges off miR-1287-5p from its target mucin 19 (MUC19) and promotes cancer cell growth, invasion, and migration through elevated glycolysis *in vitro* and *in vivo* (76). Hypoxia also induces circDENND4C expression in a HIF1α-dependent manner to promote proliferation in breast cancer cells (77), though downstream molecular mechanisms remain to be elucidated. Overall, multiple miRNAs, lncRNAs, and circRNAs directly contribute to cancer proliferation and progression downstream of HIF-signaling.

#### 2.2.2 Evasion of apoptosis

Urothelial cancer associated 1 (UCA1) is a hypoxia-responsive lncRNA whose expression is upregulated in breast cancer tissues. It is primarily transcribed by HIF1α and promotes cell proliferation by inhibiting apoptotic cell death under hypoxic conditions, though downstream molecular mechanisms are not established yet (78). Alternatively, hypoxia induces the lncRNA nuclear paraspeckle assembly transcript 1 (NEAT1) in a HIF2α-dependent manner and reduces apoptotic cell death in breast cancer cells. Elevated NEAT1 expression in tumor tissues is associated with poor survival in breast cancer patients. Mechanistically, NEAT1 controls gene regulation through the formation of paraspeckle nuclear structures, leading to nuclear retention of certain proteins and RNAs in hypoxic environment. Notably, mRNA transcripts of junction adhesion molecule 1 (JAM1) are among targets of HIF2α/NEAT1-mediated gene regulation (79). Hypoxia induced miR-424 promotes resistance to chemotherapies such as doxorubicin and etoposide by targeting the apoptosis-related tumor suppressor programmed cell death 4 (PDCD4) in breast cancer cells (80). In addition to regulating HIF1α expression through inhibition of FIH1, miR-24 further aids cancer cells by targeting pro-apoptotic BCL2-like 11 (BCL2L11, Bim) to promote resistance towards chemotherapy-driven apoptosis in breast CSCs (41). These studies highlight that hypoxia aids cancer cells to evade apoptosis by regulating multiple ncRNAs downstream of HIF-signaling.

#### 2.2.3 Angiogenesis

In order to access nutrients in bulk, cancer cells tend to initiate angiogenic molecular reprogramming under hypoxic conditions, which not only helps these cells to survive but also aids in the metastatic spread of cancer (81). The hypoxic core of tumor tissue may have differential expression of certain ncRNAs compared to the outer edges, which have an abundance of oxygen. For instance, miR-374a is lower in the center of tumor mass compared to the edges and may be associated with differential and opposite expression of its angiogenic targets VEGFA and vascular cell adhesion molecule 1(VCAM1) in tumor mass (82). Similarly, miR-20a and 20b are also differentially distributed within breast tumor mass, along with the opposite expression patterns of HIF1α and VEGFA in these tumors (83). miR-20b may inhibit VEGFA transcription and translation by simultaneously inhibiting HIF1α, STAT3, and VEGFA expression, thereby decreasing the nuclear accumulation of the HIF1α/STAT3 complex on the VEGFA promoter as well (84). VEGFA is also a direct target of miR-503. HBXIP regulates VEGFA expression and angiogenesis in breast cancer cells by inhibiting miR-503 expression as well as by hyperactivating PI3K/Akt-mediated HIF1α signaling (85). Upon hypoxia, HIF2α specifically induces transcriptional expression of the lncRNA RAB11B-AS1, which favors migration and invasion of cancer cells by inducing angiogenesis. Mechanistically, this lncRNA promotes the recruitment of RNA-polymerase II on the promoters of angiogenic genes such as VEGFA and angiopoietin-like 4 (ANGPTL4) (86). As mentioned earlier, hypoxia-driven ER stress induces miR-153 expression-mediated downregulation of HIF1α to fine-tune VEGFA expression and angiogenesis in TNBC (50), but this axis is counteracted by hypoxia mediated induction of the lncRNA HIF1α-AS2, which targets miR-153 and promotes angiogenesis (51). *In vitro* 3D culture studies illustrate that hypoxia drives vasculogenic mimicry in cancer cells to form channel-like networks resembling endothelial blood vessels. Independent studies show that miR-204 may inhibit the formation of such vascular channels by targeting focal adhesion kinase (FAK) (87) and CAMP responsive element binding protein 5 (CREB5) (88) under hypoxia. Notably, hypoxia counteracts the effects of miR-204 through the induction of the lncRNA named HOX transcript antisense RNA (HOTAIR), which induces angiogensis in breast cancer cells by sponging-off miR-204 from its targets (87). Certain cancer cells may express the hemostatic glycoprotein Von Willebrand factor (VWF) as well for their own benefits (89). Interestingly, hypoxia-induced Yin and Yang 1 (YY1) transcription factor works as a transcriptional activator of VWF, which in turn promotes angiogenesis and breast cancer cell migration. Although miR-424 works as a negative regulator of VWF expression and post-transcriptionally represses it, hypoxic environment downregulates miR-424 as well to further enhance the expression of VWF and associated angiogenic response (90). Furthermore, cancer cells tend to release excessive amount of exosomes under hypoxic conditions to communicate with surrounding cells. Hypoxic cancer cells preferentially release miR-210 loaded exosomes (91), which can work on other normoxic cancer cells within the TME, and promote VEGFA expression and angiogenesis by targeting vascularization modulators such as Ephrin A3 (EFNA3) and protein tyrosine phosphatase non-receptor type 1 (PTP1B) (92). These findings suggest that HIF-signaling-driven ncRNAs boost angiogenic signaling in the tumor that in turn promotes breast cancer cell survival.

#### 2.2.4 EMT and metastasis

With increasing hypoxia as tumors grow in size and become more aggressive, cancer cells tend to undergo EMT, intravasate into the circulatory system, and extravasate to distant organs to develop metastatic tumors. HIFs and associated signaling mechanisms are already established as key players regulating breast cancer EMT and metastasis (93). In order to fine-tune global miRNA expression patterns, hypoxia silences DICER promoter by inhibiting H3K27me3 demethylases KDM6A/B resulting in reduced miRNA processing. In particular, EMT-related transcription factor zinc finger E-box binding homeobox 1 (ZEB1) gets upregulated due to repressed expression of miR-200 family miRNAs (37). Under hypoxia, HIF1α directly inhibits miR-338-3p transcription, leading to increased expression of its downstream target and EMT-related transcription factor, ZEB2, which then promotes cancer progression, EMT, and metastasis in an NF-kB and PI3K/Akt signaling-dependent manner (94). HIF1α also promotes metastasis in breast cancer through inhibiting miR-140-5p, which regulates NRF2/H-O1 signaling by directly targeting NRF2 expression. Notably, NRF2 may directly regulate HIF1α expression to promote hypoxia and subsequent tumor growth, angiogenesis, invasion, and metastasis in a feedback loop manner (61). JNK signaling stabilizes HIF1α expression to promote transcription of another EMT-related transcription factor, TWIST1, which then transcribes miR-10b to inhibit epithelial markers and escalate EMT and metastasis. Cellular Communication Network Factor 5 (CCN5) may fine-tune this signaling by inhibiting JNK signaling at the top (95). Hypoxia-driven preferential splicing of the methyl-CpG binding domain protein 2 (MBD2) gene to the MBD2a isoform promotes breast cancer metastasis. Mechanistically, HIF1α upregulates miR-222 expression, which in turn inhibits serine and arginine rich splicing factor 2 (SRSF2), the gene controlling alternative splicing of MDB2 transcripts, leading to preferential MDB2a production. As a result, MDB2a transcriptionally activates frizzled class receptor 1 (FZD1) expression and promotes EMT and metastasis in breast cancer cells (96).

Stress, driven by a lack of nutrients, induces the unfolder protein response (UPR) or endoplasmic reticulum (ER) stress response, which is mediated through the ER-localized transmembrane sensor IRE1 and its substrate XBP1. Interestingly, XBP1 interacts with HIF1α and assembles a transcriptional complex to regulate a set of genes, which promote cancer progression in TNBC (97). Notably, MALAT1 is one of the key targets at the downstream of the XBP1-HIF1α axis whose expression correlates with aggressive phenotype, and occurrence of metastasis in breast cancer patients through regulating MYC expression (98). Interestingly, HIF2α, along with HIF-independent mechanisms, also establishes chromatin interactions at the MALAT1 promoter to regulate its transcription and subsequent metastasis in breast cancer cells (99). HIF1α also induces lncRNA induced by hypoxia and abundant in TNBC (lncIHAT) expression to promote cancer cell survival and lung metastasis *in vivo*. This lncRNA then works in a cis-acting manner and promotes the expression of neighboring oncogenes, including pyruvate dehydrogenase kinase 1 (PDK1) and integrin subunit alpha 6 (ITGA6) (100). HIF1α-AS2 is upregulated in breast cancer tissue compared to adjacent normal tissues and is associated with poor clinical outcome in patients entailing advanced tumor grade, lymph node metastasis, and distant metastasis, along with shorter overall survival (101). Hypoxia also induces transcription of multiple lncRNAs from the EFNA3 locus in HIF-dependent manner, which post-transcriptionally stabilizes EFNA3 and aids in cancer cell extravasation into distant sites to develop metastatic tumors (102). LncRNA small nucleolar RNA host gene 1 (SNHG1) is also regulated by hypoxia. HIF1α may promote SNHG1 expression, which then binds to miR-199a-3p, rescuing its post-transcriptional target TFAM, and leads to breast cancer cell metastasis (103). LINC00926 is associated with good clinical outcome and survival in breast cancer patients. It suppresses proliferation, migration and invasion of breast cancer cells by inhibiting phosphoglycerate kinase 1 (PGK1) expression *via* promoting STUB1-driven ubiquitination of PGK1. Unfortunately, LINC00826 is downregulated in breast cancer tissue compared to healthy controls, and this downregulation is attributed to hypoxia-driven inhibition of forkhead box O3 (FOXO3A) dependent transcription of LINC00926, which results in advanced tumor growth and lung metastasis *in vivo* (104). Hypoxia induced circular RNA circDENND4C targets well established tumor suppressor miRNAs, miR-200b and miR-200c (105, 106), and sponges them off of their known targets to promote glycolysis, migration, invasion, and metastasis in breast cancer cells (107). Overall, these studies highlight the role of hypoxia-driven ncRNAs in facilitating breast cancer cells to undergo EMT and metastasize to distant organs.

#### 2.2.5 Therapy resistance

Therapy resistance revolves around the limited intake of drug transport into cancer cells, the acquisition of mechanisms to get rid of the drug, and the activation of alternate signaling cascades to counteract the therapeutic effects of drug (108). Hypoxia inhibits miR-873-5p, thereby promoting the expression of its post transcriptional targets such as multi-drug resistance 1 (MDR1) and pregnane X receptor (PXR), which are involved in resisting drug influx into cancer cells (109). Hypoxic tumor microenvironment leads to inhibition of miR-326 expression in a HIF1α-dependent manner in cancer cells, in turn, promoting ITGA5 expression and hyperactivation of subsequent integrin signaling, resulting in chemotherapy resistance in TNBC cells (110). In hypoxic TNBC cells, HIF1α also drives lysyl oxidase (LOX) expression to regulate the tumor microenvironment *via* downstream integrin/FAK signaling and, as a result, confers resistance to chemotherapeutic agents. Here, HIF1α directly regulates LOX at the transcriptional level as well as inhibits miR-142-3p expression, which has the potential to simultaneously target HIF1α, LOX, and ITGA5 (111). Hypoxia induces docetaxel resistance in TNBC cells by inhibiting miR-494 in a HIF1α-dependent manner, thereby rescuing Survivin from miR-494 mediated degradation. Pharmacological targeting of HIF1α or overexpressing miR-494 sensitizes TNBC cells to docetaxel treatment *in vivo* (112). In addition, Hypoxia-induced miR-424 targets the apoptosis-related tumor suppressor PDCD4, rendering breast cancer cells resistant to chemotherapies such as doxorubicin and etoposide. Notably, miR-424 expression negatively correlates with that of PDCD4 in clinical samples of breast cancer as well (80). In *in vitro* multicellular tumor spheroids, hypoxia-induced lncRNA methionine adenosyl transferase 2 non-catalytic beta subunit (lncMAT2B) promotes cisplatin resistance by elevating DNA damage repair (113). Tamoxifen treatment in ER+ breast cancer cells may induce lncRNA UCA1 expression in a HIF1α-dependent manner, leading to tamoxifen resistance overtime. Mechanistically, UCA1 promotes hypoxic signaling by masking miR-18a mediated degradation of HIF1α through sponging off miR-18a, rendering miR-18a unable to inhibit HIF1α and its other targets involved in cell cycle progression (114). These findings suggest that hypoxia-driven inhibition of tumor suppressor ncRNAs and upregulation of oncogenic ncRNAs play crucial roles in conferring resistance to different therapeutic agents in breast cancer cells (**Table 1**).

**Table 1:** List of hypoxia-driven ncRNAs with key targets and mechanisms through which resistance to different therapeutic agents is acquired upon hypoxia.

Hypoxia-driven ncRNAs	Up/Downregulated	Targets/mechanisms	Resistance to	Ref
miR-873-5p	Down	MDR1	Drug influx	(109)
miR-326	Down	ITGA5	Doxorubicin Paclitaxel	(110)
miR-142-3p	Down	HIF1α, LOX, ITGA5	Doxorubicin Paclitaxel	(111)
miR-494	Down	Survivin	Docetaxel	(112)
miR-424	Up	PDCD4	Doxorubicin Etoposide	(80)
lncMAT2B	Up	DNA damage repair	Cisplatin	(113)
UCA1	Up	miR-18a	Tamoxifen	(114)

#### 2.2.6 Stemness

Tumor heterogeneity, attributed to varying degrees of tumor-initiating potential or stemness among cancer cells, facilitates adapting to changing tumor microenvironment, maintaining tumor aggressiveness, and resisting therapeutic interventions. Hypoxia is now recognized as a key contributing factor to cancer stemness (115). For instance, hypoxia is shown to induce embryonic stem cell markers such as OCT4, NANOG, SOX2, KLF4, and cMYC, along with miR-302, in HIF-dependent manner in different cancer cell lines, including those of breast origin (116). Hypoxia-driven miR-210 expression is now well-established as a key regulator of metabolism, cell cycle progression, proliferation, angiogenesis, DNA damage response, and apoptosis in multiple cancer types, including breast cancer (117, 118). This miRNA also promote breast cancer cell stemness through targeting E-cadherin, which is also involved in limiting EMT and metastasis (119). Notably, breast CSCs overexpress the E12/E47 transcription factor to transcriptionally regulate miR-495 expression, which then promotes cell proliferation *via* DNA damage inducible transcript 4 (REDD1) inhibition and cell invasion *via* E-cadherin inhibition (120). Carbonic anhydrase IX (CAIX) is a hypoxia marker involved in pH regulation to facilitate cancer cells’ adaptation to the changing environment during hypoxic shift. Interestingly, CAIX enriched cancer cells exhibit CSC markers. Mechanistically, CIAX fine-tunes the double negative feedback loop between LIN28 and let-7 miRNA in favor of LIN28 overexpression, leading to elevated glycolysis through increased expression of PDK1 and PDH, contributing to the acquisition of CSC markers such as NANOG and ALDH1 (121). In addition, lncRNA H19 also targets miRNA let-7 under hypoxic conditions, leading to the release of HIF1α from let-7-mediated suppression, resulting in activation of PDK1 downstream and increased stemness in breast cancer cells (122). HIF1α transcriptionally regulate lncRNA HCG18 to promote cancer stemness and proliferation. Mechanistically, HCG18 sponges off miR-103a-3p from its target ubiquitin-conjugating enzyme E2O (UBE2O). UBE2O progressively promotes cancer growth and progression *via* upregulating AMPK/mTORC1, which induces HIF1α, completing a positive feedback loop to confer a continuous hypoxic environment favoring cancer cell progression and stemness (123). Hypoxia induced Runx family transcription factor 2 (RUNX2) transcriptionally activates the lncRNA RBM5-AS1 which is involved in maintaining breast cancer stemness through stabilizing the β-catenin-TCF4 transcriptional complex by preventing β-catenin degradation (124). Hypoxia-induced lncRNA KB-1980E6.3 is related to poor prognosis in breast cancer patients. It stabilizes c-Myc mRNA by recruiting insulin-like growth factor 2 mRNA-binding protein 1 (IGF2BP1) and promotes Myc signaling mediated self-renewal in breast CSCs (125). Stromal cells such as tumor associated fibroblasts may induce TGFβ signaling in breast cancer cells to hyperactivate the peroxisome proliferator activated receptor alpha (PPARα)/HIF1α axis, which regulates multiple survival and self-renewal genes including CAIX, apolipoprotein E (APoE), snail family transcriptional repressor 2 (Slug) and interleukin 6 (IL6) to promote cancer stemness. Although PPARγ can inhibit the PPARα/HIF1α axis through upregulating miR-17-5p expression, miR-130b is induced under hypoxia to counter the inhibitory effects of PPARγ on the PPARα/HIF1α axis (126). These findings suggest that HIF-signaling-driven ncRNAs aid cancer cells to acquire stem cell like characteristics.

#### 2.2.7 Immune evasion

Hypoxia regulates immune suppression through the induction of miR-25 and miR-93 in a HIF1α independent manner. Upregulation of these miRNAs under hypoxia establishes an immunosuppressive environment characterized by an increased number of Treg cells, and a decreased population of CD8+ effector T cells. At the molecular level, these miRNAs are induced in a Tet methylcytosine dioxygenase 1 (TET1)-dependent manner and target the lysine acetyltransferase NCOA3 to inhibit transcription of cyclic-GMP-AMP synthase (cGAS), which works as a critical factor for the innate immune system in detecting cytosolic DNA. Notably, upregulated miR-25 and miR-93 expression inversely correlates with cGAS expression in clinical tumor samples and is associated with poor clinical outcome in breast cancer patients (127).

## 3 Clinical implications of hypoxia-driven ncRNAs in breast cancer

Owing to the fact that ncRNAs are often expressed in tissue- and cancer-type specific manners, tumor tissue-specific differential expression of ncRNAs may serve as prognostic biomarkers in different cancers (128). In relation to this, miR-210 is extensively studied as a hypoxia-driven prognostic ncRNA in breast cancer. It is regulated in HIF1α-dependent manner and correlates well with the hypoxia signature in breast cancer samples. In addition, it is a prognostic factor for poor disease-free and overall survival in breast cancer patients (129). Hypoxia-induced miR-210 may also serve as an independent prognostic factor in TNBC, as it is highly expressed in TNBCs compared to other subtypes of breast cancer. Moreover, it is also associated with poor overall and disease-free survival in TNBC patients (130). Hypoxia induced miR-210-3p may work as a predictive biomarker for docetaxel treatment in breast cancer as it is found to be associated with the worst outcome and poor survival in patient in response to docetaxel treatment in sequential therapy with anthracyclines (131). Furthermore, quantifying exosomal miRNA levels may also work as diagnostic tool as hypoxia not only increases the release of exosomes but also of miR-210 loaded exosomes from breast cancer cells (91). Single nucleotide polymorphisms in miRNA genes (miRSNPs) may profoundly alter global transcriptome compared to genetic polymorphisms in single gene and play an important role in different aspects of cancer biology. For instance, altered targeting of HIF1αN due to miRSNPs in the let-7 family miRNA is associated with predicting complete pathological response towards taxane and platinum based chemotherapeutic treatment in breast cancer patients (132). The hypoxia-related gene signature of twelve different lncRNAs is also proposed as a prognostic model to predict clinical outcomes such as therapeutic response, immune cell infiltration and survival in breast cancer patients (133). In addition, another set of four hypoxia-related lncRNAs is shown as a powerful tool to predict immune cell infiltration, and overall survival in breast cancer patients (134). Moreover, higher expression of hypoxia related circRNF20 aligns well with increased tumor volume and lymphatic metastasis, and predicts poor overall survival in breast cancer patients (55).

The most extraordinary aspect of ncRNAs is their presence in the circulatory system, which has led to their utilization as diagnostic and prognostic biomarkers in detecting cancers, monitoring the disease course, and assessing the therapeutic response over time. Due to their high stability and abundance, clinical quantification of circulating ncRNAs serves as an efficient, non-invasive tool for providing information about tumor growth and treatment efficacy (135). Only a few hypoxia-driven circulatory ncRNAs are reported in breast cancer so far. For instance, a bi-miRNA signature comprising hypoxia-driven oncogenic miR-210 and tumor suppressor miR-152 is proposed as a diagnostic biomarker of breast cancer. In addition, miR-152 plasma levels correlate well with different stages of the disease (136). Similarly, a tri-ncRNA signature comprising AS-ncRNA in the INK4 locus (ANRIL), HIF1α-AS2, and UCA1 may serve as diagnostic biomarker for TNBC as these lncRNAs are frequently upregulated in the plasma of TNBC patients compared to non-TNBC patients (137). Overall, hypoxia-driven ncRNAs, whether tumor-specific or circulating ncRNAs, are potential candidates to be utilized as diagnostic and prognostic biomarkers of disease progression and therapeutic response in breast cancer.

## 4 Targeting hypoxia-driven ncRNAs in breast cancer

Given their central role behind cancer progression in solid tumors, such as breast cancer, directly targeting HIFs provides an excellent therapeutic option as a treatment for solid tumors in clinical settings (138–140). The scientific community has extensively invested over the years and utilized multiple approaches such as inhibition of HIF mRNA expression, protein synthesis, dimerization, DNA binding, and transcriptional activities to identify potent HIF inhibitors. As a result, multiple HIF targeting inhibitors are now being tested in clinical trials to be approved for commercial use alone or in combination with other therapeutic regimens against multiple advanced stage cancers. Notable mentions include 2ME2 NCD (panzem) that inhibits HIF protein synthesis and transcriptional activity, EZN-2208 that suppresses HIF1 mRNA expression, Vorinostat (SAHA) and antibiotic (geldanamycin)-derived 17-AAG which increase HIF degradation, and HIF2α antagonists PT2385 and PT2977 (141). Targeting downstream effectors of HIF signaling is another promising approach to countering overall hypoxia-driven cancer aggressiveness (142). For instance, limiting hypoxia-driven secretion of heat shock protein 90α (HSP90α) by targeting a specific 115-amino acid long peptide is proposed to inhibit distant metastasis in breast cancer (143). Though still in infancy, a few studies have explored the ways to target hypoxia-driven ncRNAs in breast cancer and have shown that disrupting ncRNA mediated adaptive response to hypoxia my aid in directing hypoxic signaling to work in favor of killing cancer cells. No doubt, miR-210 is the most studied miRNA downstream of hypoxic signaling in cancer. Notably, a small molecule named Targapremir-210 that attaches to the DICER binding sites of pre-miR-210 leading to inhibited production of mature miRNA, resulting in upregulation of its targets. Glycerol-3-phosphate dehydrogenase 1-like enzyme (GPD1L) is identified as a target that not only promotes HIF1α degradation at the upstream through inhibiting PHDs but also triggers apoptosis in hypoxic breast cancer cells both *in vitro* and *in vivo* (144). Moreover, a small molecule named “1” inhibits the production of miR-544, specifically under hypoxic conditions. Mechanistically, this molecule directly targets DICER and DROSHA binding sites in pre-miR-544 transcripts, leading to depression of its mRNA targets, and hyperactivates mTOR signaling downstream, resulting in chemosensitivity and apoptotic cell death (145). Advances in nanotechnology based drug delivery options have greatly facilitated the devising of therapeutic strategies to achieve targeted delivery of ncRNAs to cancer cells (146). For instance, combined delivery of tumor suppressor miR-205 and metastasis suppressor miR-182 along with the chemotherapeutic agent doxorubicin using RNA hydrogel-based nanoparticles alleviates hypoxia-driven TNBC progression and metastasis (147). Hypoxia-driven downregulation of miR-340-5p promotes EMT and metastasis in breast cancer cells by upregulating E3 ubiquitin ligase SIAH2, which directly stabilizes HIF1α. Sinomenine, a herbal alkaloid, upregulates miR-340-5p expression and exerts its anticancer activities through inhibiting the SIAH2/HIF1α axis downstream (64). Despite recent advances in the utilization of ncRNA-based therapies, targeting hypoxia-driven ncRNAs in cancer, in general, and in breast cancer, in particular, is still evolving and awaits further biological insights to reach from bench to bedside.

## 5 Challenges and future prospect

Hypoxia-driven non-coding genome is expanding exponentially, with a lot of different ncRNAs, including miRNAs, lncRNAs, circRNAs being identified as downstream target of hypoxia or HIF-signaling, which also actively contribute to hypoxia-driven cancer progression (148–150). Of note, advances in computational pipelines to detect ncRNAs are playing a pivotal role in providing novel insights into the biogenesis and function of ncRNAs under hypoxic stress (151). Although extensive use of genome-wide approaches to identify ncRNA transcribing regions in the genome is underway, a lot of ncRNAs are still hiding out there in our genome and need to be explored for their contribution to biological processes (152, 153). Moreover, the biological functions of certain ncRNAs, such as piRNAs, snRNAs, and snoRNAs, are still underexplored in cancer biology (30). Therefore, we are still midway towards identifying the true transcriptional spectrum of hypoxia in cancer as well as towards understanding the true nature of ncRNAs in cellular mechanisms, in general, and in hypoxia, in particular. Another level of complexity in understanding the role of hypoxia-driven ncRNAs in breast cancer aggressiveness arises from the fact that certain ncRNAs may regulate and/or get regulated by HIF signaling simultaneously. On top of that, hypoxia may regulate the expression of different ncRNAs in a HIF-independent manner as well.

Tissue-specific expression and circulating levels of certain ncRNAs are now being utilized in clinics for the diagonosis and prognosis of diseases (30, 154). Similarly, tumor tissue specific expression or circulating levels of a bunch of hypoxia-driven ncRNAs are already proposed to be used as diagnostic and prognostic markers of disease progression, tumor stage, survival, therapeutic response, and immune cell infiltration in breast cancer. Of note, hypoxia-driven miR-210 is a prime candidate to become a standard in identifying the extent of hypoxia-driven tumor aggressiveness in different cancers in addition to breast cancer (118). With the advancements in ncRNA-targeted therapies in cancer (155, 156), as well as with the improvements in nanotechnology-based techniques to efficiently deliver such ncRNAs (146), it is need of time that miR-210 targeting TargapremiR-210 also get tested in clinical settings on an urgent basis against hypoxia-driven aggressive, advanced stage, and metastatic solid cancers.

Another underexplored area in HIF biology is how these transcription factors and downstream signaling pathways may behave if activated under normoxia. Initial evidence suggests that miR-101-mediated downregulation of VHL induces HIF1α-driven cell cycle arrest and apoptosis in cancer cells under normoxia (157). It is in part in line with the findings that HIFs may upregulate the expression of tumor suppressor lncRNA mitochondrial oxygen-responsive transcript 1 (MTORT1), which sponges off miR-26a-5p from its targets CREB1 and serine/threonine kinase 4 (STK4), and inhibits cell growth and proliferation in breast cancer cells (158), in contrast to the well-established tumor promoting functions of HIFs under hypoxic conditions. Therefore, exploring the downstream effectors of HIFs, including both protein-coding and ncRNAs, under normoxia as well as modulating HIFs under normoxia may also advance an alternative way to tackle tumor aggressiveness in cancer, in general, and in breast cancer, in particular.

## 6 Conclusion

Hypoxia is now established as the driving force behind tumor aggressiveness, leading to therapy resistance, metastasis, and stemness in solid tumors, including those of breast cancer. With the great advancements in exploring the regulatory roles of non-coding genome in recent years, hypoxia-responsive genome now encompasses multiple types of ncRNAs, such as miRNAs, lncRNAs, and circRNAs. These RNAs are often transcriptionally regulated downstream of HIFs and contribute to orchestrating aggressive hypoxic tumor environment by working as downstream effectors in cellular processes such as proliferation, evasion of apoptotic death, extracellular matrix remodeling, angiogenesis, migration, invasion, EMT, metastasis, therapy resistance, stemness, and evasion of the immune system in breast cancer. Alternatively, hypoxia drives the expression of certain ncRNAs in a HIF-independent manner as well, which may eventually work as upstream modulators of HIF signaling in breast cancer. Interplay between ncRNAs as well as feedback and feedforward loops between ncRNAs and HIFs further adds to the complexity of understanding hypoxia-driven ncRNAs in cancer, in general and in breast cancer, in particular. Certain hypoxia-driven ncRNAs are already proposed to be used as diagnostic and prognostic markers of disease course in breast cancer. Overcoming the challenges in devising ncRNA-targeted therapies, especially the development of efficient delivery systems in the future, will efficiently aid in the utilization and targeting of hypoxia-driven ncRNAs to treat aggressive hypoxic breast cancer in clinics.

## Author contributions

HHA, KRZ, XX and QH wrote the manuscript. HHA prepared figures. UR reviewed the manuscript. QH and TZ devised the layout and supervised the manuscript. All authors read and approved the final manuscript.
